# Midterm risk of cancer with metal-on-metal hip replacements not increased in a Finnish population

**DOI:** 10.1080/17453674.2018.1487202

**Published:** 2018-06-18

**Authors:** Elina Ekman, Inari Laaksonen, Antti Eskelinen, Pekka Pulkkinen, Eero Pukkala, Keijo Mäkelä

**Affiliations:** 1 Department of Orthopaedics and Traumatology, Turku University Hospital, Turku;; 2 Coxa Hospital for Joint Replacement, Tampere;; 3 Department of Public Health, Helsinki University, Helsinki;; 4 Faculty of Social Sciences, University of Tampere, Tampere;; 5 The Finnish Cancer Registry, Institute for Statistical and Epidemiological Cancer Research, Helsinki, Finland

## Abstract

Background and purpose — Metal-on-metal (MoM) total hip arthroplasty (THA) and hip resurfacing arthroplasty (HRA) have been widely used during the early 21st century. We assessed the midterm risk of cancer of patients treated with modern MoM hip implants compared with patients with non-MoM hip implants and the general Finnish population with special interest in soft tissue sarcomas and basalioma due to the findings of our previous report.

Patients and methods — All large-diameter head MoM THAs and hip resurfacings performed in Finland between 2001 and 2010 were extracted from the Finnish Arthroplasty Register (10,728 patients). Patients who underwent conventional THA formed the non-MoM reference cohort (18,235 patients). Data on cancer cases up to 2014 were extracted from the Finnish Cancer Registry. The relative risk of cancer in the general population was expressed as the ratio of observed to expected number of cases, i.e., standardized incidence ratio (SIR). Poisson regression analysis was used to compare the cancer risk between the cohorts. The mean follow-up was 7.4 years (1–14) in the MoM cohort and 8.4 years (1–14) in the non-MoM cohort.

Results — The overall risk of cancer in the MoM cohort was comparable to the general Finnish population (SIR 0.9, 95% CI 0.9–1.0). Risk of basalioma in the MoM cohort was higher than in the general Finnish population (SIR 1.2, CI 1.1–1.4) and higher than in the non-MoM cohort in the stratified regression analysis (RR 1.2, CI 1.0–1.4, p = 0.02). The SIR of soft-tissue sarcoma in the MoM cohort was 1.4 (CI 0.6–2.8); the incidence was same as in the non-MoM cohort.

Interpretation — Metal-on-metal hip implants are not associated with an increased overall risk of cancer during midterm follow-up.

Second-generation large-diameter head (LDH) MoM THA and HRA gained popularity at the beginning of the 21st century (FAR, AOANJRR 2016, NJR 2016). Metal particles emanate as a result of corrosion and wear of metal-on-metal (MoM) hip implants and can disseminate throughout the body. These particles can be found in several organs including lymphatic tissue, bone marrow, liver, and spleen (Case et al. 1994, Shea et al. 1997, Urban et al. 2000, Shimmin and Back 2005). Metal debris from hip implants has been associated with chromosomal aberrations and DNA damage (Case et al. 1996, Bonassi et al. 2000, Daley et al. 2004, Polyzois et al. 2012, Sarhadi et al. 2015). Wear particles are released both from MoM and conventional metal-on-polyethylene (MoP) bearings and from the trunnion of MoM THA (Pastides et al. 2013). However, the risk of cancer was not increased after conventional MoP THA or after earlier used first-generation MoM THA (Visuri et al. 1996, 2010).

Previous studies have found no increase in the overall risk of cancer after second-generation MoM hip arthroplasty in the short term when compared with other bearing types (Mäkelä et al. 2012, Smith et al. 2012, Brewster et al. 2013, Lalmohamed et al. 2013). However, follow-up time in these studies is relatively short as cancer takes years to develop. A recent meta-analysis could not find causative relationship between second-generation MoM implants and cancer risk (Christian et al. 2014). At the same time, a previous Finnish study reported an increased incidence of basalioma and soft tissue sarcoma in patients treated with MoM implants compared with patients treated with non-MoM implants (Mäkelä et al. 2014). Sarcoma is a severe, life-threatening disease and due to these earlier findings we felt obligated to update our data with longer follow-up.

We have now updated our earlier results on risk of cancer (Mäkelä et al. 2012, 2014) in patients treated with primary MoM hip implants compared with patients treated with primary non-MoM THAs and the general Finnish population in midterm follow-up, specifically the risk of sarcoma, basalioma, and skin melanoma.

## Patients and methods

In Finland virtually all cancer cases are recorded in the population-based Finnish Cancer Registry (Teppo et al. 1994, Pukkala et al. 2018) and 98% of primary total hip implants are recorded in the Finnish Arthroplasty Register (FAR). LDH MoM THAs and hip HRAs performed in Finland between 2001 and 2010 were extracted from the FAR and formed the MoM cohort. Patients who underwent metal-on-polyethylene, ceramic-on-polyethylene, or ceramic-on-ceramic THA during the study period formed the non-MoM reference cohort. All of these study subjects were followed-up until December 31, 2014 for emigration and vital status via the Population Registry, and for cancer incidence via Finnish Cancer Registry through a personal identity code. None of the patients were lost to follow-up. While forming the study population we included the first hip implant in every patient. If a patient received another hip implant later, only those who had similar bearings in both sides were included.

There were 10,728 patients in the MoM cohort and 18,235 patients in the non-MoM THA cohort included in this study; 46% of the patients were men (Tables 1 and 2). The number of person-years at follow up was 79,521 for the MoM cohort and 152,358 for the non-MoM cohort. Of all our patients 497 (4.6%) had bilateral MoM implants. The mean follow-up was 7.4 years (0–14) in the MoM cohort and 8.4 years (0–14) in the non-MoM cohort.

**Table 1.** Number of patients (n) according to age at operation, and number of person-years (PY) according to the age at follow-up. The non-metal-on-metal cohort consisted of implants with metal-on-polyethylene, ceramic-on-polyethylene, and ceramic-on-ceramic bearing surfaces

	Metal-on-metal cohort				Non-metal-on-metal cohort				Total			
	Men		Women		Men		Women		Men		Women	
Age	n	PY	n	PY	n	PY	n	PY	n	PY	n	PY
0–9	1	1	–	–	–	–	–	–	1	1	–	–
10–19	5	16	3	16	–	–	–	–	5	16	3	16
20–29	25	136	16	69	4	17	7	29	29	153	23	98
30–39	159	558	67	309	30	119	20	116	189	677	87	425
40–49	741	3,746	471	2,078	143	730	157	578	884	4,476	628	2656
50–59	2,277	11,787	1,643	8,578	850	3,880	922	4,416	3,127	15,667	2,565	12,994
60–69	2,257	19,652	1,581	14,090	2,260	13,980	2,739	16,099	4,517	33,632	4,320	30,189
70–79	763	9,403	594	6,773	3,044	25,854	5,397	39,047	3,807	35,257	5,991	45,820
80–	65	1,276	48	1,036	698	13,610	1,953	33,884	763	14,886	2,001	34,920
Total	6,293	46,573	4,423	32,948	7,029	58,190	11,195	94,168	13,322	104,763	15,618	127,116

PY: person-years

**Table 2.** Baseline characteristics for the metal-on-metal cohort and for the non-metal-on-metal cohort

Factor	Metal-on- metal cohort	Non-metal-on- metal cohort
Mean age (SD)	59 (10)	71 (9)
Number of women, n (%)	4,426 (41)	11,202 (61)
Primary osteoarthritis, n (%)	9,901 (92)	17,683 (97)
Secondary osteoarthritis, n (%)	827 (8)	552 (3)

## Statistics

For both cohorts (MoM and non-MoM) the person-years at risk were calculated within stratification of sex, calendar period (2001–05 and 2006–10), 5-year age groups, and follow-up time (< 2, 2–5, and >5 years since the operation). The expected number of each type of cancer within each stratum was calculated by multiplying the person-years in the stratum by the stratum’s age, sex, and calendar-period-specific cancer incidence rate for the Finnish population. The total expected numbers of cancers were summed over the strata. The cancer risk relative to the Finnish population, i.e., standardized incidence ratio (SIR), was expressed as the ratio of observed to expected number of cases. For the 95% confidence intervals (CI), we assumed that the number of observed cases followed a Poisson distribution. The study population and detailed information on the implant types have been presented in detail in earlier publications (Mäkelä et al. 2012, 2014).

Poisson regression analysis was used to estimate the relative cancer risk between the MoM and non-MoM cohorts for soft tissue sarcomas, melanoma and basalioma. Soft-tissue sarcoma and basalioma were chosen for Poisson regression due to the earlier results of Mäkelä et al. (2012, 2014) and skin melanoma due to earlier results with conventional THA (Visuri et al. 2003, Onega et al. 2006). The risk estimate (incidence rate ratio) was adjusted for age (0–49,50–59, 60–69, 70–79, 80+) and follow-up time (< 2, 2–5, and >5 years since the operation). Poisson regression analysis was checked for over-dispersion. The level of statistical significance was set at p < 0.05.

## Ethics, funding, and potential conflicts of interest

Ethical approval: 13.6.2017, Dnor THL/926/5.05.00/2017. This research received no funding. The authors declare no conflicts of interest.

## Results

The overall risk of cancer in patients treated with MoM hip implants was slightly lower than in the general Finnish population (SIR 0.9, 95% CI 0.9–1.0) (Table 3).

**Table 3.** Observed numbers of cancer cases, the expected numbers of cancer cases approximated from the Finnish population, and standardized incidence ratios with 95% confidence intervals—according to site—are given for the metal-on-metal cohort and for the non-metal-on-metal cohort. The latter cohort consisted of implants with metal-on-polyethylene, ceramic-on-polyethylene, and ceramic-on-ceramic bearing surfaces

	Metal-on-metal cohort					Non-metal-on-metal cohort				
Primary site	Obs	Exp	SIR	95% CI	% of cancer	Obs	Exp	SIR	95% CI	% of cancer
All sites	915	973	0.9	0.9–1.0	9	2851	2852	1.0	1.0–1.0	16
Stomach	23	21	1.1	0.7–1.7	0.2	75	74	1.0	0.8–1.3	0.4
Colon	48	55	0.87	0.6–1.2	0.4	187	199	0.9	0.8–1.1	1
Lung	61	95	0.64	0.5–0.8**^a^**	0.6	203	260	0.78	0.7–0.9**^a^**	1
Corpus uteri	24	22	1.1	0.7–1.6	0.2	78	82	1.0	0.8–1.2	0.4
Prostate	239	216	1.1	1.0–1.2	2	478	461	1.0	1.0–1.1	3
Kidney	31	29	1.1	0.7–1.5	0.3	83	82	1.0	0.8–1.2	0.5
Bladder	32	41	0.8	0.5–1.1	0.3	131	128	1.0	0.9–1.2	0.7
Soft-tissue sarcoma	8	6	1.4	0.6–2.8	0.07	20	17	1.2	0.7–1.8	0.1
Non-Hodgkin lymphoma	37	38	1.0	0.7–1.4	0.3	118	108	1.1	0.9–1.3	0.7
Hodgkin lymphoma	2	2	0.9	0.1–3.1	0.02	3	4	0.7	0.1–2.0	0.02
Multiple myeloma	13	12	1.1	0.6–1.8	0.1	36	41	0.9	0.6–1.2	0.2
Leukemia	17	18	1.0	0.6–1.5	0.2	60	58	1.0	0.8–1.3	0.3
Melanoma	38	36	1.1	0.8–1.5	0.4	105	87	1.2	1.0–1.5	0.6
Basalioma	306	246	1.2	1.1–1.4**^a^**	3	913	878	1.0	1.0–1.1	5

Obs: observed number of cancer cases; Exp: expected number of cancer cases based on cancer incidence in the comparable Finnish population; SIR: standardized incidence ratio; CI: confidence interval; % of cancer: the percentage of patients diagnosed with a certain cancer during the follow-up.

**^a^**p < 0.001

There were 8 soft-tissue sarcomas in the MoM cohort during the follow-up period (SIR 1.4, CI 0.6–2.8) (Table 2). The risk of soft-tissue sarcoma in the MoM cohort was the same than that in the non-MoM cohort (RR 0.9, CI 0.4–2.0, p = 0.8).

Incidence of basalioma in the MoM cohort was higher than in the general Finnish population (SIR 1.2, CI 1.1–1.4; p < 0.001) (Table 3) and also higher than that of the non-MoM cohort (RR 1.2, CI 1.0–1.4, p = 0.02).

The SIR of skin melanoma in the MoM cohort was 1.1 (CI 0.8–1.5) and that in the non-MoM cohort was 1.2 (CI 1.0–1.5) (Table 3). Risk of melanoma in the MoM cohort was not higher than that in the non-MoM cohort (RR 0.9, CI 0.6–1.4, p = 0.7).

## Discussion

We found that the overall midterm risk of cancer was not increased in patients treated with MoM hip implants when compared wit the general Finnish population in midterm follow-up. This is in line with previous short term follow-up studies on second-generation MoM hip implants (Mäkelä et al. 2012, Smith et al. 2012, Brewster et al. 2013, Lalmohamed et al. 2013, Mäkelä et al. 2014). The slightly lower overall risk of cancer in the MoM group can be influenced by the fact that MoM patients tend to be young and possibly healthier than the average population, which might cause some selection bias. A recent study from Slovenia including only THAs found a slightly higher risk of overall cancer in patients treated with MoM bearing when compared with the general population or the non-MoM patients (Levasic et al. 2018). In that study the specific cancer types that had higher prevalence in the MoM cohort compared with the general population were skin cancers excluding melanoma and prostate cancer. Comparably, we found higher risk for basalioma in our MoM cohort. This confirmation of our results from another country is an interesting finding, and needs further research. The study cohort size in the study by Levasic et al. was smaller than ours (338 MoM THAs). Prostate cancer risk was not increased in the MoM cohort in our study when compared with the general Finnish population.

Although MoM hip implants are associated with local pseudotumors, adverse local tissue reactions (ALTR), and possible genetic alterations, based on earlier literature it seems that the risk for systemic tumors is not elevated after MoM THA (Case et al. 1996, Pandit et al. 2008, Ollivere et al. 2009, Langton et al. 2010). Our findings are in line with these studies. Sarhadi et al. (2015) studied DNA extracted from periprosthetic tissues of 20 MoM patients undergoing hip revision surgery because of ALTR. They found genetic alterations in 6 patients and a liposarcoma in 1 patient.

In our previous short-term follow-up study of this same study population the risk of soft-tissue sarcomas was elevated in the MoM group compared twith the non-MoM-group (Mäkelä et al. 2014). Furthermore, in that study all sarcomas were diagnosed during the last 4 years of the follow-up, raising a concern that during longer follow-up soft-tissue sarcomas might be overrepresented in the MoM cohort and that there might be a causative relationship between metal wear debris and soft-tissue sarcomas. However, in the current study only 1 additional soft tissue sarcoma was observed during the additional follow-up years 2012–14, and the incidence was similar in the MoM patient population compared with the general Finnish population and similar to the risk in the non-MoM group. Nonetheless, the overall number of sarcomas is small and we plan to report further follow-up. To our knowledge, there are no other studies reporting increased incidence of soft-tissue sarcomas in patients treated with metal-on-metal hip implants.

Incidence of basalioma was higher in the MoM cohort than in the non-MoM cohort and also increased when compared with the Finnish population. A similar finding has previously been reported only with conventional total hip replacements (Brewster et al. 2013). The majority of the previous studies on MoM hip implants either exclude non-melanoma skin cancer or include basaliomas in the category of other skin cancers and the data on basalioma incidence in patients treated with MoM implants is limited (Visuri et al. 2006, Smith et al. 2012, Lalmohamed et al. 2013). Due to its benign nature, basalioma is traditionally not included in the official national cancer statistics. In Finland only the first basalioma for each person is recorded in the Finnish Cancer Registry (Pukkala et al. 2018). This may bias our results since patients treated with HRA are generally younger than patients treated with conventional THA, and it may therefore be more likely for them to be diagnosed with basalioma for the first time during our follow-up.

We found that the incidence of skin melanoma was not elevated in the MoM cohort compared with the general Finnish population. Earlier studies have found conflicting evidence concerning conventional non-MoM THAs’ association with melanoma incidence. Some studies have reported higher melanoma incidence in patients treated with non-MoM implants than in the general population (Nyrén et al. 1995, Olsen et al. 1999, Visuri et al. 2003, 2006) while others have found no difference (Visuri et al. 2010, Levasic et al. 2018). No increase in the risk of melanoma was found for patients treated with a MoM resurfacing device (Brewster et al. 2013).

The study by Brewster et al. (2013) found an increased risk of multiple myeloma and other immunoproliferative neoplasms in THA patients during the first 4 years after arthroplasty. However, their study did not differentiate MoM bearings from other types of bearings and the study also included patients with rheumatic conditions, which are known to increase the risk of immunoproliferative neoplasms (Isomäki et al. 1978). Our study found no excess risk of myeloma in the MoM hip implant patients.

We acknowledge that our study has several limitations. First, as with all registry-based studies there is a possibility of a selection bias. Registry-based studies have the advantage of reporting results from a large patient group and reporting so-called “real world data” but the disadvantage of possible confounding by indication (Freemantle et al. 2013). That is, the patients selected for THA or HRA may be for example healthier than the average population. Ideally this could be avoided by randomized controlled studies. Second, we did not have any blood metal ion measurements or imaging findings of the patients. It is theoretically possible that higher cancer risk might be associated with higher ion levels and our study is not able to detect such a subgroup. However, the findings of the meta-analysis by Christian et al. (2014) suggest that the concentrations and doses of Co/Cr required to induce a genotoxic or tumorigenic outcome are much higher than the systemic Co/Cr concentrations typically present in MoM hip implant patients. Third, even though our follow-up time now reaches the midterm point, genetic alterations might still happen or manifest later. In a recent meta-analysis Pijls et al. (2016) reported a higher risk of mortality in patients with MoM THA compared with patients with non-MoM THA when the follow-up exceeded 10 years. No difference was noted with shorter follow-up. Considering this we plan to report long-term results from this same population.

In summary, patients treated with MoM hip implants had a comparable cancer risk to patients treated with non-MoM hip implants and the general Finnish population. They did not have increased risk for soft-tissue sarcoma or skin melanoma. Only the incidence for basalioma was increased in the MoM cohort compared with the non-MoM cohort and compared with the general population.

KM designed and coordinated the study and helped to draft the manuscript. EE collected the data and drafted the manuscript. EP calculated the statistics. KM, IL, PP, AE, EP and EE contributed to the interpretation of the data and results and to the preparation of the manuscript. All authors read and approved the final manuscript.

*Acta* thanks Bart G Pijls and other anonymous reviewers for help with peer review of this study.
